# Supplementary figures and images for: Motor neuron activity enhances the proteomic stress caused by autophagy defects in the target muscle
Source: PLoS One. 2024 Jan 2;19(1):e0291477. doi: 10.1371/journal.pone.0291477 (PMC10760831; doi:10.1371/journal.pone.0291477)

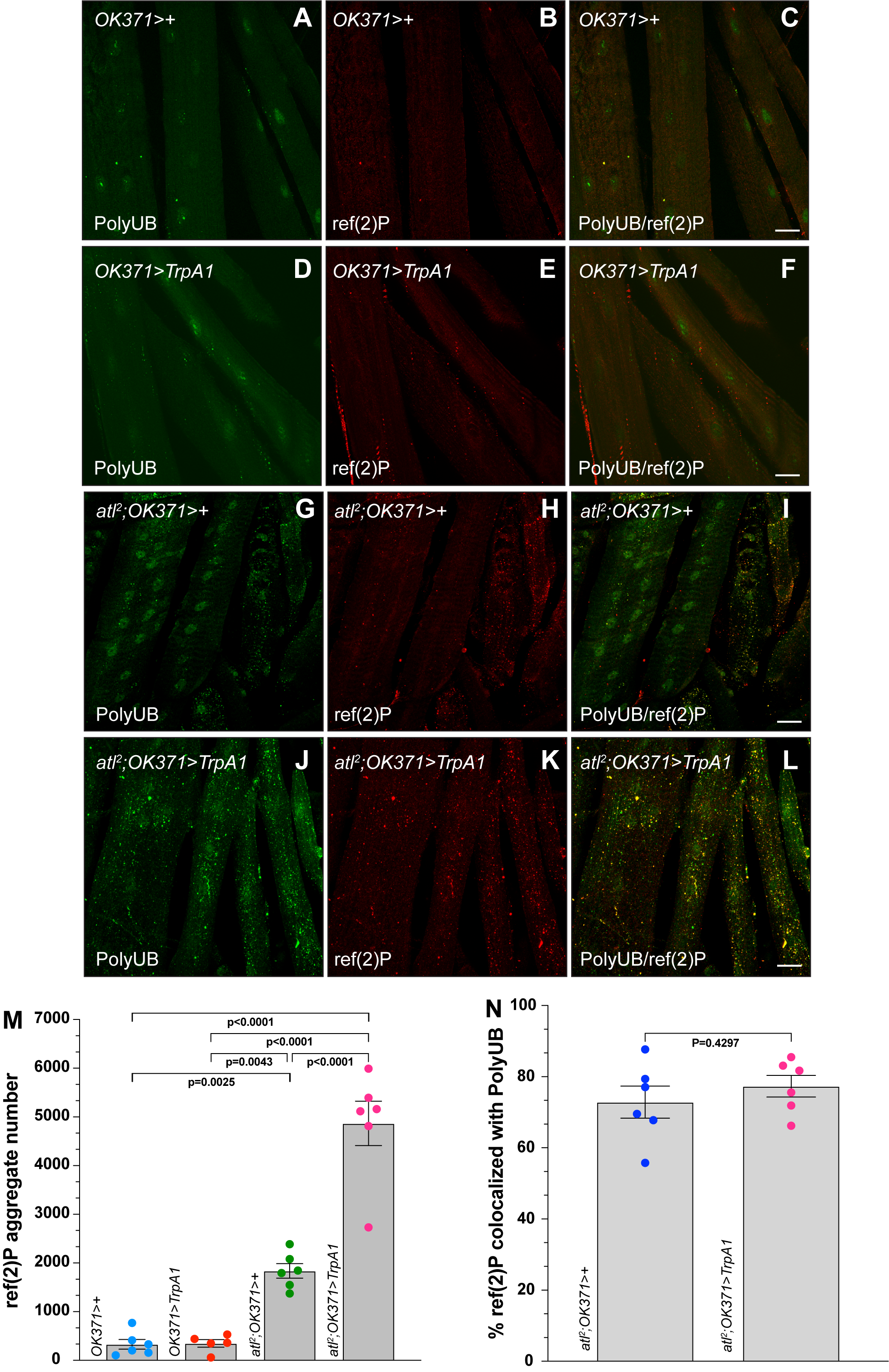

Supplement: S1 Fig — A)-L) Confocal imaging (maximum intensity z projection) of fixed third instar larval muscle cell 6 of the indicated genotypes stained with an anti-ubiquitin antibody (green, panels A,D,G,J), an anti-ref(2)P antibody (red, panels B,E,H,K) and merge (panels C,F,I,L). Scale bar 20 μm. M) Means +/- SEMs of ref(2)P aggregate number (Y axis) as a function of genotype (X axis). N) Percent of the poly-UB punctae that are also contain ref(2)P (Y axis) for the indicated genotypes. P-values were calculated by Student’s t-test. All larvae were reared at 28° C. (TIF) [file pone.0291477.s001.tif]

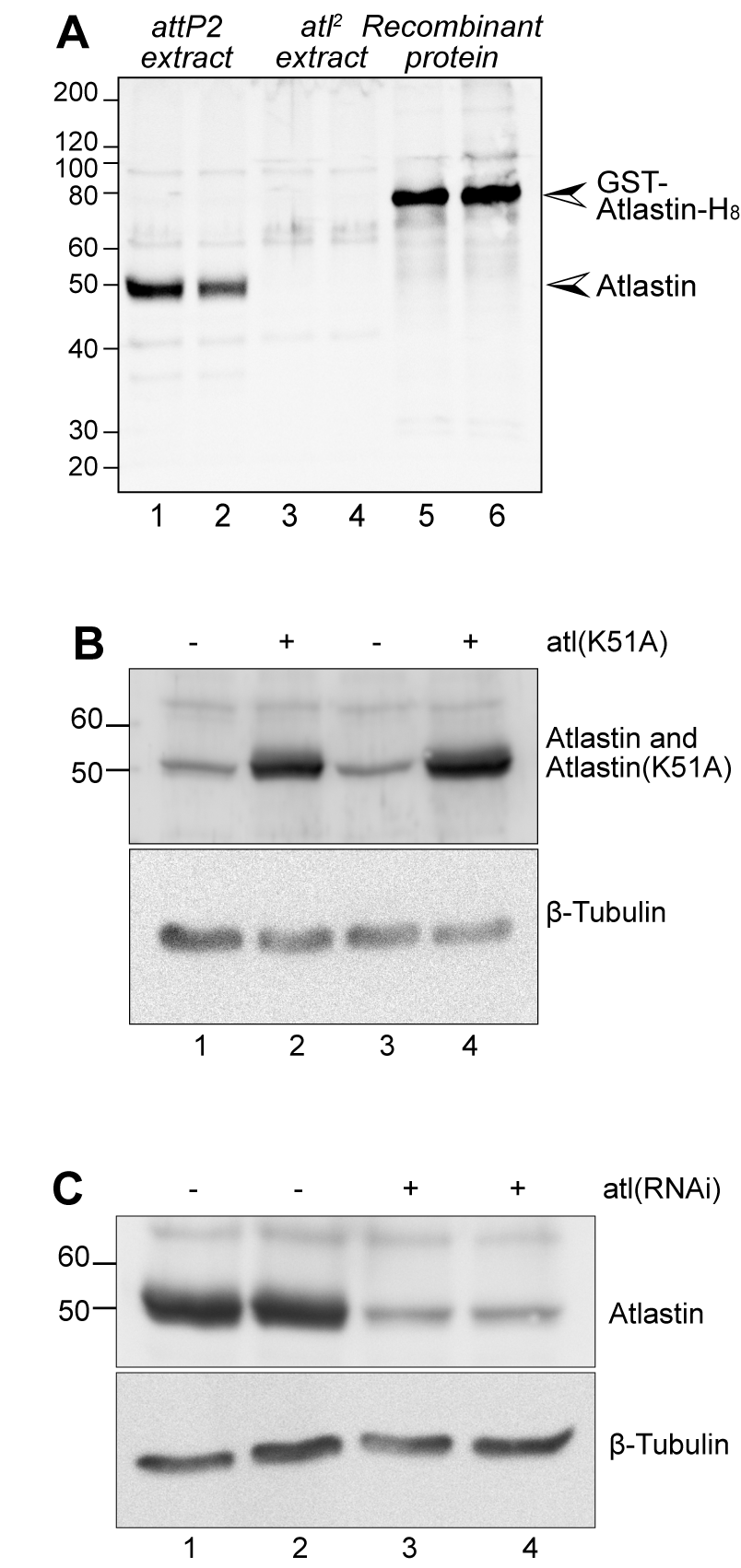

Supplement: S2 Fig — A) Western blot with anti-Atl antibody from whole fly from wildtype (attP2, lanes 1 and 2), atl2 (lanes 3 and 4), and recombinant GST and His-tagged Atl (lanes 5 and 6). B) Heads from flies carrying nSyb-lexA and either Aop-driven atlK51A (lanes 2 and 4) or empty vector (lanes 1 and 3). C) Heads from flies carrying nSyb-lexA and either empty vector (lanes 1 and 2) or Aop-driven atl RNAi (lanes 3 and 4). β-tubulin serves as loading control in B) and C). (TIF) [file pone.0291477.s002.tif]

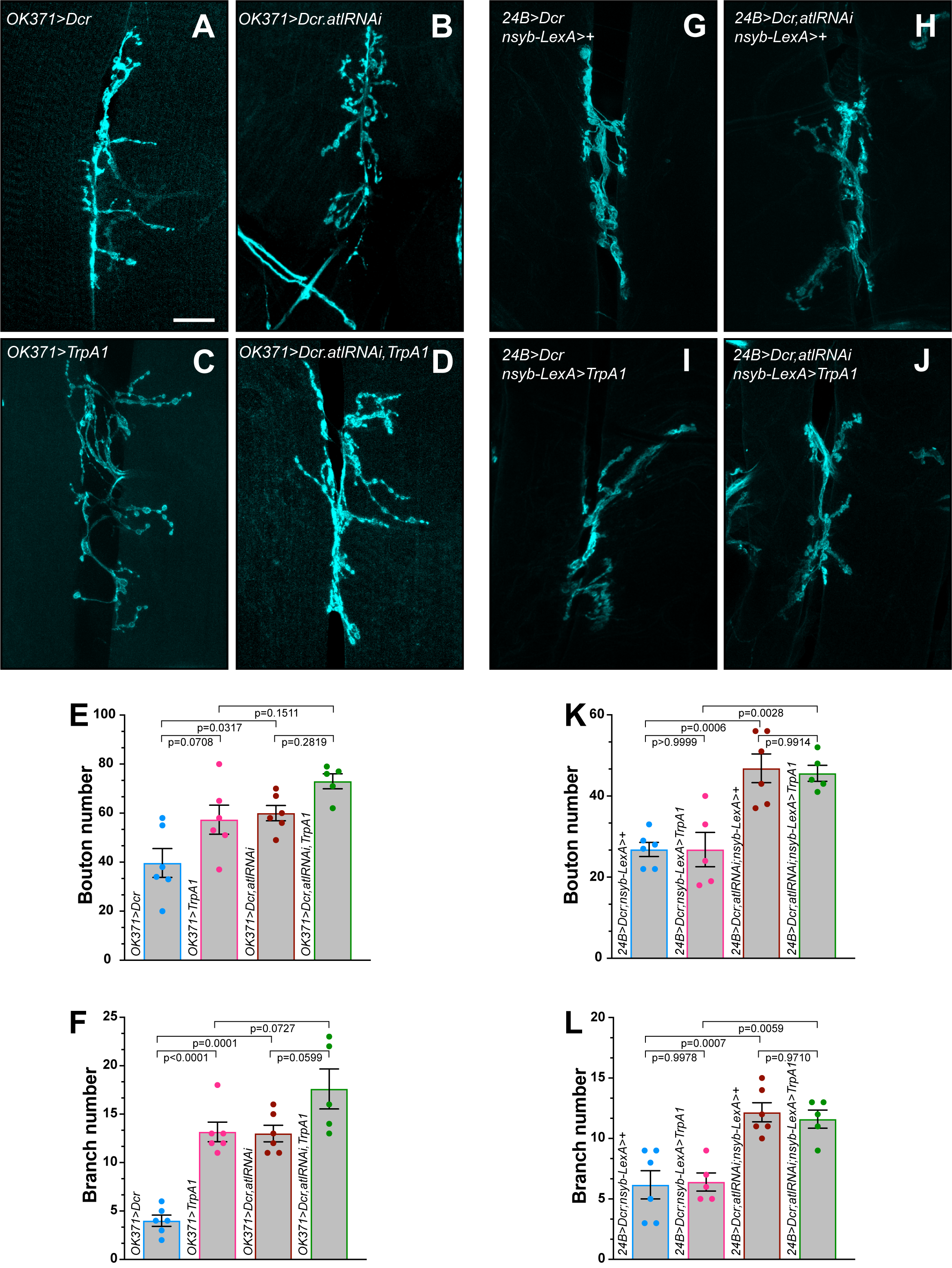

Supplement: S3 Fig — Larval nerves innervating muscles 6 and 7 were labelled with Alexa fluor 647 conjugated anti-HRP and imaged on a Zeiss LSM 800 with a 40x objective. A)-D) and G)-J) show neuromuscular junctions from larvae of the indicated genotypes, shown in pseudo cyan. E), F), K), and L) Scattergram showing means +/- SEMs of bouton number (E) and axon branch number (F) for larvae of genotypes. p values were calculated by a one-way ANOVA and Tukey’s post-hoc test. n = 5 or 6. (TIF) [file pone.0291477.s003.tif]

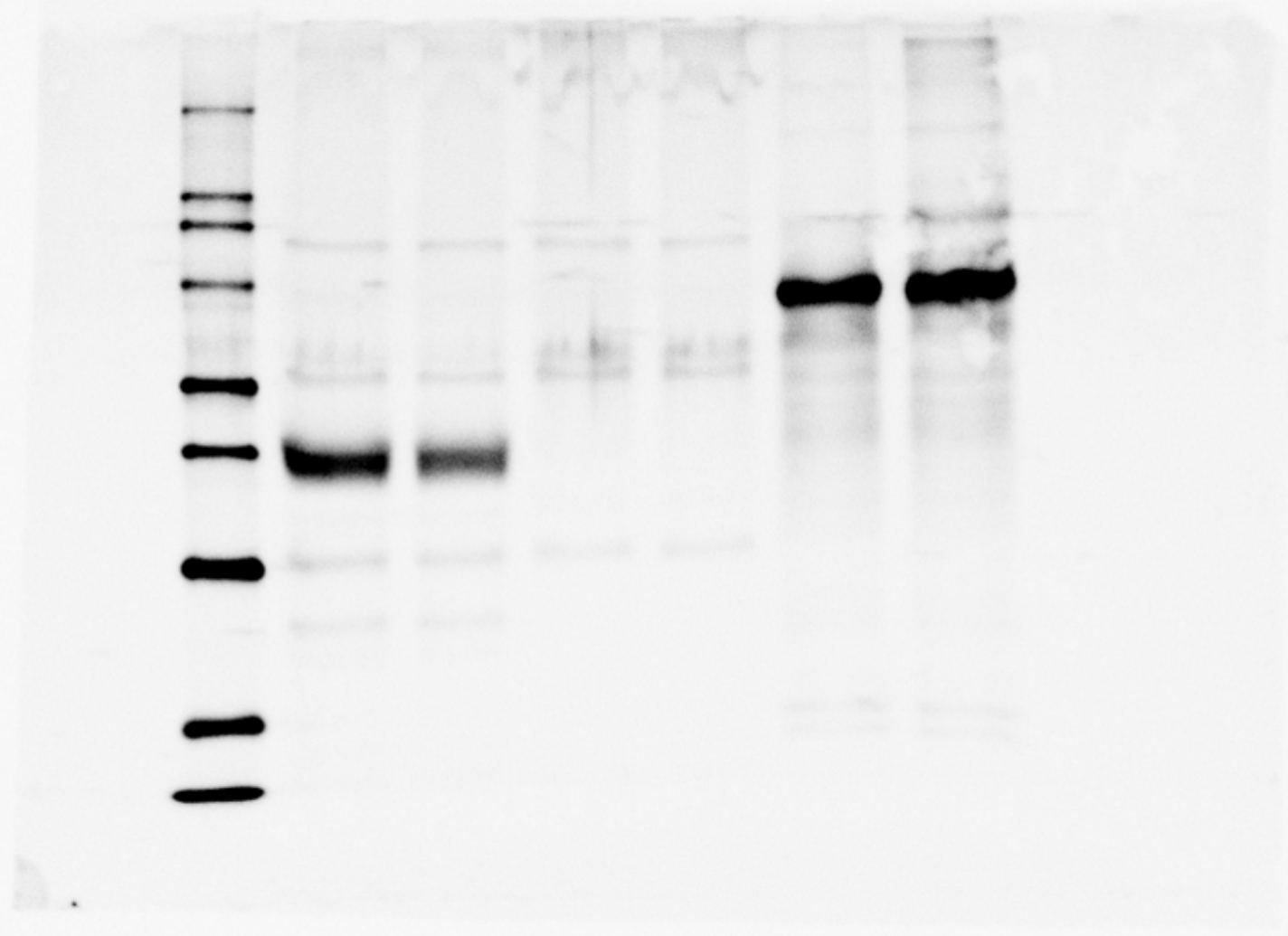

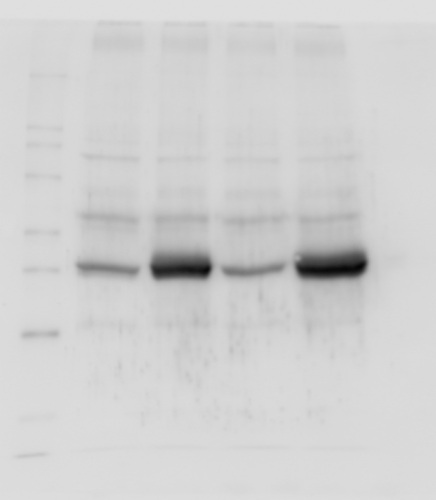

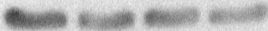

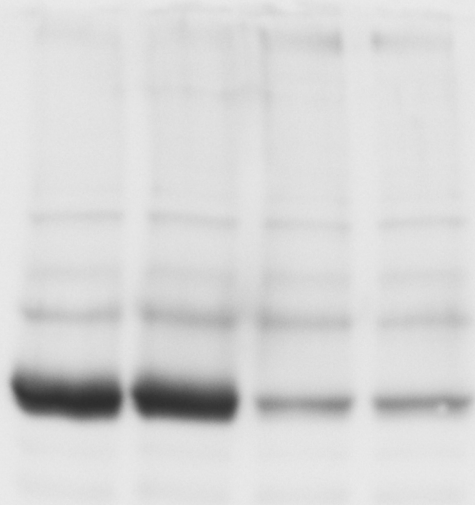

Supplement: S1 Raw images — (PDF) [file pone.0291477.s004.pdf]
